# The pattern of prognostic and risk indicators among women with breast cancer undergoing modified radical mastectomy in Dar es Salaam, Tanzania

**DOI:** 10.1186/s13027-016-0075-8

**Published:** 2016-06-30

**Authors:** Amos R. Mwakigonja, Happiness Rabiel, Naboth A. Mbembati, Leonard E. K. Lema

**Affiliations:** Department of Surgery, Muhimbili University of Health and Allied Sciences (MUHAS), Dar es Salaam, Tanzania; Department of Pathology, Muhimbili University of Health and Allied Sciences (MUHAS), Dar es Salaam, Tanzania

## Abstract

**Background:**

Breast cancer is the commonest female malignancy globally and the second (after uterine cervix) in sub-Saharan Africa including Tanzania. Prognostic indicators reportedly influence post-mastectomy adjuvant therapy by predicting risks on survival and recurrence although in Tanzania this data is lacking. Here, we evaluate the pattern of prognostic and risk indicators among women with breast cancer undergoing modified-radical-mastectomy (MRM) at Muhimbili National Hospital (MNH) and Tumaini Hospital (TH), Dar es Salaam, Tanzania.

**Methods:**

This hospital-based prospective cross-sectional study included female patients undergoing MRM from April 2011 to January 2012. Clinical stage I-III patients were enrolled after being scheduled for mastectomy. Patients with evidence of distant metastasis (stage IV) were excluded. Mastectomy and axillary lymph nodes biopsies were submitted to the Histopathology laboratory for grade, type, nodal and margins status. Data was collected using a structured questionnaire and analyzed using SPSS.

**Results:**

A total of 348 patients were admitted with breast cancer including 86 patients (with 16 from TH having similar demography and presentation) meeting inclusion criteria. Age-range at diagnosis was 28–79 years, mean 52.1 years. Most (89 %) attained menarche after 11 years. About 56 % were postmenopausal. The majority (78 %) were multiparous with positive family history in 14.1 and 37.6 % used hormonal contraceptives.

About 27.1 % were social alcohol drinkers. The majority (61 %) had T4b disease, 75.6 % had positive axillary nodes including 42.7 % with 4–9 involved nodes (N2). The commonest (91.9 %) histological type was invasive ductal carcinoma. Lobular, medullary and mucinous carcinomas were rare. Most (83.7 %) of our patients presented with stage III and the rest stage II. Intermediate- and high-grade tumors accounted for 73.5 %. Following MRM, 25 % of our patients had positive surgical margins and similarly for the base.

**Conclusions:**

Most of our breast cancer patients present with frequent risks including younger age, multiparity, hormonal contraceptives use, alcohol use and family history. Unfavourable prognostic indicators including late stages, large primary tumor size, skin infiltration, positive surgical margins, positive axillary lymph nodes and a high histological grade were associated.

A sustainable screening program by self-examination to allow early diagnosis is needed to reduce morbidity and mortality from this cancer.

## Background

Breast cancer is the most frequently diagnosed cancer globally and the leading cause of cancer death among females, accounting for 23 % of the total cancer cases and 14 % of the cancer deaths [1]. Breast cancer is the second most common malignancy among females in sub-Saharan African countries including Tanzania, the leading cancer being of that of the uterine cervix [2]. In Tanzania, the Ocean Road Cancer Institute (ORCI) is the main centre of care for cancer patients. Its 2004 registry reported admission of 10–20 patients with breast cancer per month [2].

The prognosis of patients with breast cancer is influenced by a number of factors including those which are tumor-related including the size of the primary carcinoma, lymph node involvement and the number of lymph nodes involved by metastases. Studies have shown that the presence of estrogen and progesterone receptors and currently HER2 to influence the prognosis of patients with breast cancer [3]. The presence of estrogen and progesterone receptors is important clinically as a predictor for response to adjuvant hormonal therapy rather than prognostic factors [4]. The presence of hormone receptors (ER and PR) in the tumor tissue correlates well with the response to hormonal therapy and chemotherapy [5]. HER2 over expression in patients with breast cancer has been associated with poor prognosis and resistance to treatment [6]. The histological type of the tumor is one of the important prognostic indicators in breast cancer as it influences the form of treatment. Ductal carcinoma in situ (DCIS) is a pre-invasive breast cancer that can almost always be cured by local-regional therapy. The most common histological grading system for breast cancer evaluates tubule formation, nuclear grade, and mitotic rate to divide carcinomas into well-differentiated carcinomas which have a significantly better prognosis as compared with poorly differentiated carcinomas. Moderately differentiated carcinomas initially have a better prognosis, but survival at 20 years approaches that of poorly differentiated carcinomas [7]. The clinical stage of breast cancer is among the factors influencing the prognosis of these patients. Late presentation to hospital is still a challenging factor in Tanzania where only 5.2 % patients present with Stage II disease, 57 % stage III and 37.5 % stage IV. Stage I disease patients are rarely found [8]. Studies have shown that only 5 to 12 % of Stage I/II patients die in the first 10 years after diagnosis, compared with over 60 % of Stage III patients and over 90 % of Stage IV patients. Breast cancer staging also provides valuable information about appropriate treatment options for each cancer stage [9]. Post-mastectomy radiotherapy is always recommended for patients with stage IIB disease who have four or more positive axillary nodes. Other risk factors are patient-related including age, reproductive career, and menopausal status, family history, alcohol use, tobacco smoking and use of hormornal contraceptives. It has been found that younger patients in general, have favourable prognosis as compared to older women [10, 11]. However, age under 30 years has been postulated to have a worse prognosis [12, 13].

The natural history of breast cancer influences its management in which both local and systemic control of the disease, are of great importance. Depending on the stage of the disease, management of breast cancer can be individualized [14]. This would in part depend on predisposing risks as well as prognostic factors and thus these need to be elucidated in our settings in order to inform treatment and prevention/control strategies.

The current practice of treatment of breast cancer patients at our institution depending on the stage of the disease includes neoadjuvant therapy followed by mastectomy or mastectomy with adjuvant therapy. Patients destined for recurrence can be selected for systemic adjuvant therapy, and patients who will not have a recurrence can be spared the morbidity of a treatment that offers no benefit [15]. Knowledge of the prognostic indicators of patients with breast cancer before and after surgical intervention may assist in proper management of these patients including the determination as to who will benefit from specific adjuvant therapy.

In Tanzania, studies on prognostic indicators of patients with breast cancer including late presentation, histological grade, axillary lymph node status, surgical margins and base status, presence of bilateral disease, hormone receptor status and HER2 status and others, are not yet well documented. Thus, the current study aims at enlightening surgeons, oncologists as well as pathologists and other stakeholders who care for breast cancer patients by elucidating the pattern of disease presentation within our settings so as to allow the formulation of improved as well as individualized management resulting in increased survival and quality of life. This is particularly important in a country like Tanzania where post-treatment follow-up of patients is greatly limited by logistical constraints on the part of both the patient and cancer care providers.

## Methods

### Study design

Hospital-based prospective descriptive study.

### Study area

The study was conducted in the female surgical wards and the Histopathology Unit of the Department of Laboratory Services at MNH as well as the female surgical wards at Tumaini Hospital (TH) in Dar es Salaam, Tanzania from April 2011 to January 2012. The two hospitals were chosen due to geographical proximity and to allow the use of a comparable standard type of mastectomy during the study since both hospitals use Auchincloss type. Furthermore, most surgeons operating at MNH also work at Tumaini Hospital. It is also noteworthy that most patients going to TH came from MNH but resorted to the former due to long waiting lists. Importantly also, TH accepts patients with National Health Insurance Fund (NHIF) cards, just like MNH thus they are not necessarily of different socioeconomic status.

### Treatment modalities

The current practice of treatment of breast cancer patients at MNH depends on the stage of the disease and includes neoadjuvant therapy followed by mastectomy or mastectomy with adjuvant therapy. Patients destined for recurrence can be selected for systemic adjuvant therapy, and patients who will not have a recurrence can be spared the morbidity of a treatment that offers no benefit. Such decisions are conducted at a Multidisciplinary Tumour Board at MNH every Tuesday at 2.00 pm and attended by specialists from both MNH and ORCI. It consists of Surgeons, Gynaecologists, Oncologists as well as Pathologists who form the Secretariat while Oncologists chair the Board. Breast cancer patients are therefore generally categorized as premenopausal and postmenopausal. Premenopausal would be treated with chemotherapy, surgery and radiotherapy depending on staging while postmenopausal women will be treated with hormonal therapy (Tamoxifen), surgery and radiotherapy depending on staging as well. We do not give hormonal therapy to all breast cancer patients currently but we base on the menopausal status as well as ER/PR status where the later can be/has been done. Up to now there is no routine receptor status testing in Tanzania due to cost.

### Study population and sampling

All women admitted in general surgical wards with clinical stage I to III breast cancer as classified by TNM staging system undergoing MRM from April 2011 to January 2012 were included in the study.

#### Inclusion criteria

All patients admitted in female surgical wards with clinical stage I to III breast cancer who underwent MRM from April 2011 to January 2012 were included in the study.

#### Exclusion criteria

Patients with recurrent breast cancer, those who underwent neoadjuvant therapy, Stage I-III disease without axillary dissection as well as patients with stage IV disease were excluded from the study. The patients with recurrent breast cancer and those who underwent neoadjuvant therapy were excluded since the therapy alters the primary lesion, stage III patients may be down staged to stage II. Since clinical stage was one of the prognostic indicators studied, down-staged patients were excluded to avoid getting the wrong impression of clinical stage of studied patients at presentation to hospital. Patients who did not have axillary node dissection (both stage I-III and IV) were excluded since node status was also one of the prognostic indicators studied.

### Limitations of the study

This study did not cover hormonal receptors as well as HER2 determination because this was also a time bound and very financially limited student research exercise. The money available could not support at all hormone studies. This is not only a problem for this work but for most if not all of our public funded student research activities. Furthermore, immunohistochemistry including for hormone receptors is expensive globally and is not yet routine in Tanzania. Patients are therefore generally categorized as premenopausal and postmenopausal except in situtations where hormonal receptors have been done. Premenopausal would generally be treated with chemotherapy, surgery and radiotherapy depending on staging while postmenopausal women will be treated with hormonal therapy (tamoxifen), surgery and radiotherapy depending on staging as well.

### Data sources

Histopathology reports following FNAC or incision biopsy were used to confirm diagnosis of breast carcinoma while chest X-rays and abdominal pelvic ultrasounds reports to exclude stage IV patients. Physical examination was done by HR to obtain clinical stage, assess for clinically bilateral disease and signs of metastasis (pleural effusion or ascites). Histopathology reports as well as slides following mastectomy and axillary dissection were reviewed by ARM and HR and relevant information collected.

### Biopsies

The whole breast and lymph nodes were submitted to the histopathology unit and formalin-fixed and paraffin embedded (FFPE) tissue blocks were prepared as previously described [16, 17]. Glass slides were stained with hematoxylin-eosin. The sections were reviewed to determine the histopathological type, grade, node positivity, status of surgical margins and the base. Nuclear pleomorphism, tumor differentiation (well differentiated, moderate and poorly differentiated) as well as mitotic figure counts per one high power field were used to grade the tumor, where mitotic figure counts of 1–2 was regarded as low grade, 3–5 intermediate grade and above 5 high grade.

### Histopathology

Primary histological diagnosis on hematoxylin and eosin (H & E) stained formalin-fixed paraffin embedded (FFPE) sections was done as previously described at MNH [17, 18].

### Data collection

Data was collected by HR who was a Surgery Resident at the time and two research assistants; one in the ward and another in the laboratory who took mastectomy specimens to the Histopathologist (ARM) and also collected slides in order to review the histological grades which were seldom reported on routine histopathology results. Questionnaires were filled by HR and the assistant in the ward to obtain demographic data, gynecological history and details of clinical findings of the patient like tumor size, regional node involvement, bilaterality of the disease as well as histopathological information including the type of tumor, grade, number of lymphnodes submitted, number of lymphnodes positive for tumor, surgical margins and base status.

### Data handling and analysis

Data was entered in structured questionnaires which were given serial numbers in addition to hospital file numbers to assist in keeping record in a systematic manner. The copy of histopathology report of every patient was attached to the respective questionnaire. All histopathology reports with inadequate information were identified and slide number recorded for review. Patient details and results were entered into statistical programme for social scientists (SPSS) version 17 (IBM, USA) and data and analysis was done. Fisher’s exact and t-test was used in analysis and attaining statistical significance. *P*-value of <0.05 was considered significant.

### Ethical consideration

In this study, ethical clearance was sought and obtained from the MUHAS Ethical Committee and permission to conduct the study was sought from relevant authorities at MNH. The study did not change the form of treatment planned for the patients. No individual participant data is reported in the manuscript and patient information was handled in a strictly confidential way. No personal identifiers were used. Informed consent with detail on the purpose of the study, the rights of the participant and benefits on participation was obtained in writing from prospective patients.

## Results

### General demography

Between the month of April 2011 and January 2012, a total of 348 patients were admitted with breast cancer including 86 patients (out of this 16 came from Tumaini Hospital) with breast carcinoma stage I-III who underwent modified radical mastectomy (MRM) and thus met the inclusion criteria. The age range was 28–79 years; with mean age at diagnosis of 52.08 years. The peak age-group was 36–55 years and accounted for 54.6 % of patients studied. The majority were indigenous Africans with only about 1.2 % Caucasians. This was expected because in Tanzania, indigenous Africans are the majority. Among the study population, 5.8 % were smokers and 26.7 % were social alcohol drinkers. The demography and clinical presentation of patients from TH were comparable to those of MNH.

Eighty nine (89 %) of study population attained menarche after the age of 11 years, with mean age at 14.4 years. Fifty seven (57 %) were postmenopausal with 24.5 % attaining menopause at the age of 55 years or more. About 79 % were multiparous, with only 7 % nulliparous.

Most (87.5 %) of them had their first live birth before the age of 30 years. Family history of breast cancer was reported in 14 % of patients and 37.2 % reported use of hormonal contraceptives.

### Prognostic indicators and histopathology

**Fig. 1:**
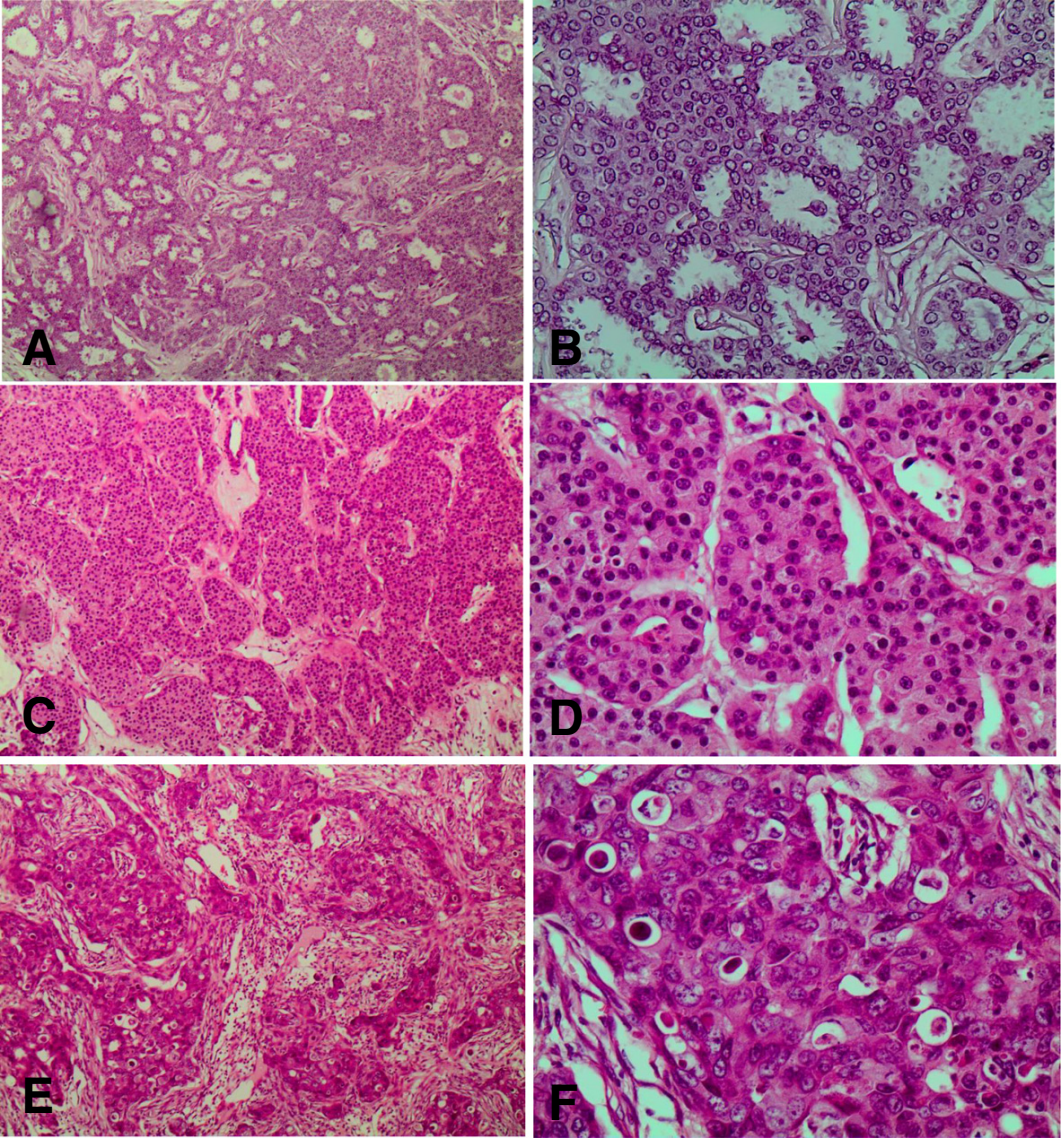
**a**: Histological section showing a well-differentiated ductal carcinoma of the breast (x 100). **b** Histological section showing a well-differentiated ductal carcinoma of the breast (x 400). **c** Histological section showing a moderately-differentiated ductal carcinoma of the breast (x 100). **d** Histological section showing a moderately-differentiated ductal carcinoma of the breast (x 400). **e** Histological section showing a poorly-differentiated ductal carcinoma of the breast (x 100). **f** Histological section showing a poorly-differentiated ductal carcinoma of the breast (x 400)

**Fig. 2:**
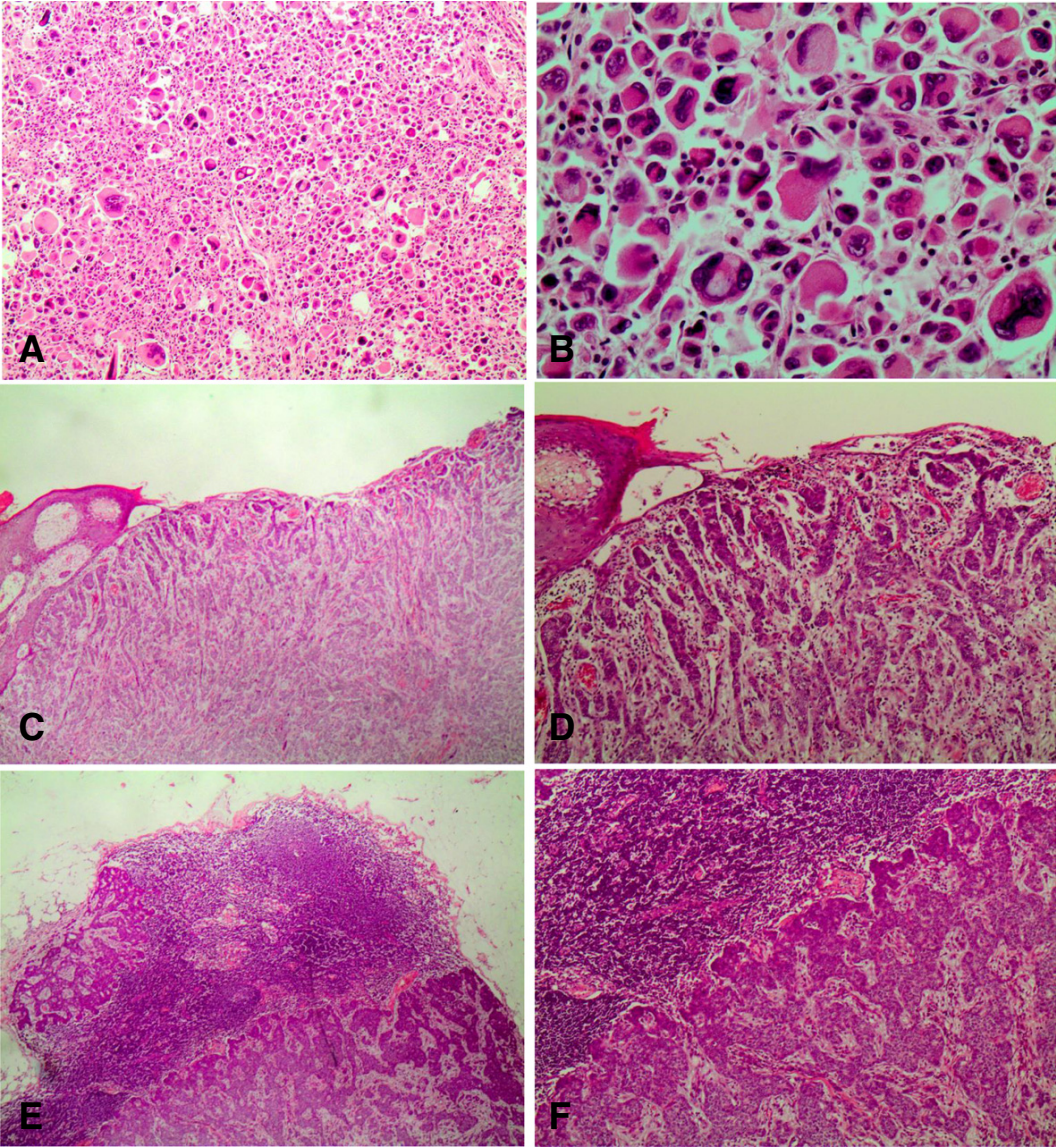
**a** Histological section showing a pleomorphic carcinoma of the breast (x 100). **b** Histological section showing a pleomorphic carcinoma of the breast (x 400). **c** Histological section showing a cutaneous involvement (Ulceration) by carcinoma of the breast making it stage T4 (x 100). **d** Histological section showing a cutaneous involvement (Ulceration) by carcinoma of the breast making it stage T4 (x 400). **e** Histological section showing a lymphnode involvement by carcinoma of the breast (x 100). **f** Histological section showing a lymphnode involvement by carcinoma of the breast (x 400)

A great majority (83.7 %, *n* = 72/86) were in clinical stage III and the majority (42.7 %, *n* = 35/86) of patients also had high (4–9) number of positive lymph nodes. Furthermore, most (73.5 %, *n* = 61/86) patients had high or intermediate histological grades as compared to those with low grade [Table 1, Fig. 1 (a-f)]. Those tumors with high grade included those with pleomorphic histology [Fig. 2 (a & b)]. The majority (60.5 %, *n* = 52/86) of women in our study had T4 tumors and none-had T1 (Table 1). T4 tumors included those with large size of primary tumor as well as those with skin involvement including ulceration [Fig. 2 (c-f)]. The findings of our current study imply that our patients mostly present with advanced disease (Table 1). Infiltrating ductal carcinoma was the most common (91.9 %, *n* = 79/86) histological type of tumor seen, while infiltrating lobular carcinoma accounted for 2.3 %. And medullary breast carcinoma was seen in 1.2 % [Table 1, Fig. 1 (a-f)]. Other histological types included both infiltrating ductal and lobular occurring in conjunction, metaplastic carcinoma, mucinous carcinoma and apocrine carcinoma constituting 4.7 %.

**Table 1:** Prognostic indicators for breast carcinoma in the study population

Prognostic indicator	Number of patients No.(%)
Clinical Stage	
Stage I	0 (0)
Stage II	14 (16.3)
Stage III	72 (83.7)
Positive axillary nodes	
0	20 (24.4)
1–3	21 (25.6)
4–9	35 (42.7)
>10	6 (7.3)
Histological Grade	
High	34 (41)
Intermediate	27 (32.5)
Low	22 (26.5)
Histological type	
Invasive Ductal	79 (91.9)
Invasive Lobular	2 (2.3)
Medullary	1 (1.2)
Others	4 (4.7)
Primary tumor	
Tx	2 (2.3)
T1	0 (0)
T2	18 (20.9)
T3	14 (16.3)
T4	52 (60.5)

**Fig. 3:**
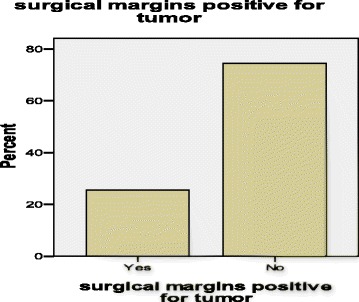
A bar chart showing the percentage tumor positivity in surgical margins among post-MRM female breast cancer patients at MNH and Tumaini Hospital

Following modified radical mastectomy, 25 % of the patients had tumor positive side surgical margins and 75 % had margins free from tumor (Table 2, Fig. 3). Interestingly, the majority (70 %, *n* = 14/20) of patients with a positive tumor base also had positive side surgical margins (*p*-value 0.026, statistically significant) [Table 2, Fig. 3] while most (84.2 %, *n* = 16/19) of our patients without lymph node involvement had negative side surgical margins with only 15.8 % positive margins (*p*-value 0.000, highly statistically significant) [Table 3, Fig. 3]. This implies that tumors which were not locally invasive were also not likely to metastasize.

**Table 2:** The association between the side surgical margins and the base of the tumor

Side surgical margins positive for tumor	Base positive for tumor		
	Yes (%)	No (%)	Total (%)
Yes	14 (70)	6 (10)	20 (25)
No	6 (30)	54 (90)	60 (75)
Total	20 (100)	60 (100)	80 (100)

Seventy percent (70 %) of patients with positive tumor base had positive side surgical margins (*P*-value 0.026, statistically significant)

**Table 3:** The association between side surgical margins and lymph node status

Margins positive	Lymph node status No. (%)		
	Positive	Negative	Total
Yes	17 (27.9 %)	3 (15.8 %)	20 (25 %)
No	44 (72.1 %)	16 (84.2 %)	60 (75 %)
Total	61 (100 %)	19 (100 %)	80 (100 %)

Most of our patients with lymph node negative disease had negative surgical margins (side) (84.2 %) with only 15.8 % positive margins (*P*-value 0.000, highly statistically significant)

Furthermore, increased age at diagnosis appeared to be associated with larger primary tumor size of T3 and T4 (*p*-value 0.049). The association between the patient’s age at diagnosis and histological grade of tumor was not statistically significant.

## Discussion

The clinical management of breast cancer patients at MNH and Tumaini Hospital (source hospitals) involves surgical treatment of patients followed by adjuvant radiotherapy/chemotherapy at ORCI (destination hospital). Patients are further followed up at ORCI clinics and will only return to MNH in case of local recurrence requiring surgical treatment or when they have complications of distant metastases such as pleural effusion requiring underwater seal drainage. This protocol hinders surgeons at source hospitals from following up these patients to understand how they fare after treatment at the destination hospital. It thus becomes difficult to establish the prognosis and survival of patients with breast cancer treated at MNH as well as Tumaini Hospital.

In breast cancer some factors have been reported to constitute risks to developing the disease; including early age at menarche, late menopause, delay in first live birth, family history as well as use of hormonal contraceptives [19, 20].

In this study, 89.4 % of study population attained menarche after the age of 11 years, with mean age of 14.3 years which is the same as what was reported from Tanzania previously [8]. Furthermore, young age at diagnosis has been associated with aggressive disease and poor prognosis [21]. A retrospective study done in Tanzania among females with breast cancer recorded in the Cancer Registry between 1974 and 1987 suggested that there was a younger age of onset among Tanzanians and in those from other sub-Saharan African countries, Latin America and Asia when compared with those from North America/Northern Europe [2, 22–24]. Furthermore, the age at menarche as well as that at first childbirth indicates a long reproductive career as influencing the risk of breast cancer in our settings. The age range of 28–79 years also suggests that we are seeing them at younger ages. This is also supported by the mean age of 52 years in our study which is much lower than that of 64 years reported among African Americans previously [25]. The mean age seems to be much lower in West Africa as reported previously and all this suggests that probably the biological behavior and pathogenesis of breast cancer in Africa could be different from that in Western industrialized countries [25, 26].

Regarding other socio-demographic factors influencing breast cancer risk and prognosis, the finding that our cohort included a majority of postmenopausal differed from that in Nigeria probably due to ethno-demographic and geographic as well as other factors [27]. Importantly, menopause status is an indicator of prognosis where premenopausal women appear to have a more unfavourable prognosis than their postmenopausal counterparts [22, 28]. Furthermore, our results agree with Nigerian and other studies on parity and alcohol consumption as being associated with breast cancer [29–31]. Likewise, our finding of a significant number of hormonal contraceptives users among our cohort agrees with previous reports [32]. Conversely, cigarette smoking did not seem to be an important characteristic among our breast cancer patients as the case could be elsewhere [25, 31].

Our current finding of a positive family history of breast cancer of 14 % in our cohort appears to be much higher than those reported from Nigeria [26, 33] and generally indicates that it is an important risk and prognostic factor in our settings. A Cypriot study found that family history is the strongest predictor of breast cancr risk in their population [19]. Furthermore, in the present study, the size of primary tumor appeared to increase with increasing age at diagnosis while patients with node positive disease were diagnosed at an earlier age than those who were negative. The variation of age with tumor size is expected as it takes a while for tumors to grow while lymphnode involvement at an earlier age implies increased aggressiveness. Thus, the larger primary tumor size and frequent tumor infiltration of the skin in our current study representing a high (T4) stage is reflective of advanced disease at diagnosis. This is most likely due delayed hospital presentation in part due to the behaviour of breast cancer which is painless initially, ignorance, seeing traditional healers before consulting modern medical care and a poorly organized referral system.. This frequent T4 stage at diagnosis is well in agreement with a previous Tanzanian report showing the mean tumor size to be 8 cm and none of them was less than 2 cm. Moreover, 68.8 % of the tumors in the same study exceeded 5 cm in their greatest diameter [8]. These findings are in contrast to a previous report from Cuba where T1 and T2 disease were more frequent (49.2 and 45.7 %) respectively [34]. The difference could be due to better existing screening programs in Cuba resulting in diagnosis at an earlier stage with smaller primary tumors size. It is noteworthy that tumor size has been found to be the strongest predictive factor for relapse even in node negative patients [35]. Thus, the findings of our study regarding primary tumor size indicate increased likelihood patients relapsing and therefore less favourable prognosis.

In the current study, the majority of patients had tumor positive axillary nodes. Axillary lymph node status is the most important prognostic factor in breast carcinoma and prognosis worsens with increasing number of metastatic lymph nodes [36]. According to the American Joint Committee on Cancer (AJCC)/International Union against Cancer (UICC) tumor (T)-node (N)-metastasis (M) classification, nodal disease is classified in three groups based on the number of axillary metastatic lymph nodes: N1, 1–3 metastatic lymph node(s), N2, 4–9 metastatic lymph nodes and N3, 10 or more metastatic lymph nodes [37]. In our index study, 42.7 % of patients had N2 disease which is an unfavourable prognostic indicator. Our findings are in contrast with those reported by a study done in Cuba where 46.6 % of tumors examined were node-negative (N0), 25.7 % had N1 disease, and 27.4 % had N2 disease [34]. The differences could in part be due to better screening programmes in Cuba thus breast cancer is diagnosed early before developing metastases. Since studies have shown increased tumor recurrence among patients with metastatic axillary nodes of up to 70 % within 10 years after mastectomy [38]. Most of patients treated at MNH and Tumaini Hospital for breast cancer are likely to have poor prognosis due to high number of metastatic axillary nodes.

As expected, in the current study, most women had invasive ductal carcinoma. This is quite in concordance with many previous studies reporting similar histological findings including a study in Japan which found invasive ductal carcinoma in 90 % of patients with breast cancer, 5 % had invasive lobular carcinoma, 1.5 % medullary carcinoma and 3 % mucinous type [39]. In Nigeria, comparable findings were reported where most (95 %) patients had infiltrating ductal carcinoma and 5 % were papillary, lobular or unspecified types [33]. These findings of our current study imply that most of patients in our studied institutions have an increased risk to poor prognosis because of the dominant histological tumor type.

Furthermore, in our index study intermediate and high histological grades were commoner than low grade tumors and these findings are in line with what was reported among Cuban women who underwent surgery for breast cancer, whereby 54.87 % were intermediate grade and 30.82 % were high grade tumors [34]. Similarly, an Irish study involving 293 cases of invasive primary breast cancer, showed that 53 % of cases had low and intermediate grades together, while 47 % had high grade tumor, further implying that higher grades as being more frequent had intermediate been grouped with high grade which by itself is almost half the proportion [40]. As expected, higher grades have been quite consistently associated with lower long-term survival as well as with increasing tumor size [28, 41]. On the other hand, there was no association between mean age at diagnosis with the histological grade of tumor. The higher histological grades seen in the index study are proportional the frequency of patients with large primary tumor size which is usually associated with higher histological grades. This implies that about 73 % of patients undergoing MRM at our institutions are likely to have a relapse due to the histological grades of their tumors.

Clinical stage of the disease at time of diagnosis can significantly affect the prognosis and outcome of the patient. Most (83.5 %) of patients in the present study had stage three disease, 16.5 % stage two and none had stage one. Ten years ago, a pathological study of surgical specimens of patients with breast cancer was done at MNH; 5.2 % were stage II and 57 % stage III, none were stage I disease [8]. In Ibadan Nigeria, a study involving 763 cases of breast cancer showed 2 % had stage I disease, 13 % stage II and 46 % stage III [38]. Conversely, in Canada most patients are seen at early stage of disease, thus in one study 37 % had stage I disease, 38.5 % stage II and 7.1 % stage III disease [42]. Among Cuban women, breast cancer was as well diagnosed at early stage compared to Tanzanian, 23 % stage I, 59.94 % stage II and 15.3 % stage III disease [34]. Clinical stage II and III disease are considered high risk stage for relapse as well as overall survival. The late presentation seen in our study population may be due to lack of well established and comprehensive screening programs as well as myths on breast cancer surgery and adjuvant therapy which are linked to death.

A tumor positive surgical margin is definitely a risk factor for local recurrence in breast cancer. In our present study, 25 % of patients had positive surgical margins of which 70 % had both base and side margins positive for tumor. Considering that a large number of patients in our cohort presented with a locally advanced disease, it was not expected that the frequency of positive surgical margins would be so low and the reasons for this discrepancy are not yet clear. Adjuvant local radiotherapy and systemic therapy may limit tumor recurrence but not to levels observed in patients with negative margins [43–45].

## Conclusions

This study has found that at the time of diagnosis, most of our patients appear to have unfavourable prognostic factors leading to decreased survival including locally advanced tumors, a younger presenting age, frequent intermediate-to-high tumor grades as well as larger primary tumor sizes. Majority of our patients were postmenopausal which seemd to be the only indicator of favourable prognosis in our index study. Furthermore, risk factors including long reproductive career, multiparity, use of hormonal contraceptives, alcohol consumption and a positive family history appeared to be relatively frequent among breast cancer patients in our cohort although larger samples and randomized studies are needed to ascertain this.

However, following mastectomy the percentage of positive margins is low thus less likelihood of local recurrence when margin status alone is taken into consideration. Thus, the majority of our patients may not require local radiotherapy after mastectomy. We recommend sustainable and comprehensive screening programs, at least by increased awareness and self-examination in our settings. Further tests including mammography, breast ultra-sound scan and fine-needle aspiration cytology which are more expensive can be reserved for those found to have suspicious lumps. Early definitive diagnosis as well as timely and good surgical intervention will also improve breast cancer outcome in Tanzania.

## Abbreviations

HER-2: human epidermal growth factor receptor-2
MRM: modified radical mastectomy
MNH: Muhimbili National Hospital
ORCI: Ocean Road Cancer Institute
TH: Tumaini Hospital
TNM: Tumor, Node (lymphnode), Metastasis (distant metastasis)
